# Evaluation of aerodigestive disease and diagnosis of sliding hiatal hernia in brachycephalic and nonbrachycephalic dogs

**DOI:** 10.1111/jvim.16485

**Published:** 2022-07-05

**Authors:** Elizabeth Luciani, Carol Reinero, Megan Grobman

**Affiliations:** ^1^ Department of Clinical Sciences Auburn University College of Veterinary Medicine Auburn Alabama USA; ^2^ Department of Medical Sciences University of Wisconsin School of Veterinary Medicine Madison Wisconsin USA; ^3^ Department of Veterinary Medicine & Surgery University of Missouri Veterinary Health Center Columbia Missouri USA

**Keywords:** brachycephalic, dysphagia, radiographs, respiratory, videofluoroscopic swallow study

## Abstract

**Background:**

Aerodigestive diseases (AeroD), hybrid disorders between the respiratory and gastrointestinal (GI) tracts, may present without GI signs. Sliding hiatal hernia (sHH) is an important AeroD in brachycephalic dogs linked to respiratory pathology. The spectrum of other AeroD and respiratory clinical signs (CS) in brachycephalic and nonbrachycephalic dogs with sHH is unknown.

**Objectives:**

Characterize CS of AeroD in dogs with sHH, compare CS between brachycephalic and nonbrachycephalic dogs, and compare thoracic radiographs and videofluoroscopic swallow study (VFSS) for diagnosing sHH.

**Animals:**

Sixty‐seven client‐owned dogs with sHH.

**Methods:**

Medical records of dogs with sHH presented to the veterinary teaching hospitals at Auburn University and the University of Missouri between 1 January 2009 and 31 December 2020 were retrospectively reviewed. Between group, comparisons were made using Mann‐Whitney test, Chi‐square analysis, and Spearman correlation (*P* < .05).

**Results:**

Dogs with sHH presented with exclusively GI signs (28/67), mixed respiratory and GI signs (22/67), or with exclusively respiratory signs (17/67). Wheras brachycephalic dogs were not significantly more likely to present with respiratory CS (*P* = .145), they were younger (*P* < .001), and more likely to present in respiratory distress (*P* = .02), and with radiographic evidence of aspiration pneumonia (*P* < .001) compared to nonbrachycephalic dogs. Six of 12 dogs with normal thoracic radiographs having sHH presented with respiratory CS. For detection of sHH, VFSS was superior to radiographs (*P* < .001).

**Conclusions and Clinical Importance:**

Dogs with sHH may present with exclusively respiratory signs. Respiratory signs may be more severe in brachycephalic compared to nonbrachycephalic dogs. Videofluoroscopic swallow study was superior to thoracic radiographs for detection of sHH in dogs.

**Abbreviations**
AARS: aspiration‐associated respiratory syndromes
AeroD: aerodigestive disease
AP: aspiration pneumonia
AU‐CVM: Auburn University
BOAS: brachycephalic obstructive airway syndrome
CS: clinical signs
GERD: gastroesophageal reflux disease
GI: gastrointestinal
EERD: extraesophageal reflux disease
IQR: interquartile range
MSBC: mainstem bronchial collapse
sHH: sliding hiatal hernia
MU‐VHC: University of Missouri Veterinary Health Center
VFSS: videofluoroscopic swallow study

## 1 INTRODUCTION

Aerodigestive diseases (AeroD) are important hybrid disorders between the respiratory and gastrointestinal (GI) tracts and reflect complex, coordinated events required for breathing and swallowing. Aerodigestive diseases are known contributors to respiratory clinical signs in both people and dogs. Some examples of AeroD include brachycephalic obstructive airway syndrome, aspiration pneumonia and aspiration pneumonitis, diffuse aspiration bronchiolitis, gastroesophageal reflux disease (GERD), and extraesophageal reflux disease (EERD, defined as refluxate extending beyond the esophagus to contact structures of the upper airway causing pathology).^1^, ^2^, ^3^, ^4^, ^5^, ^6^, ^7^, ^8^ Importantly, AeroD may present in the absence of GI clinical signs (CS) and with normal thoracic radiographs making clinical recognition challenging.^2^ Correction of the underlying AeroD has been demonstrated to improve clinical outcome, making identification of AeroD critical for optimal case management.^6^, ^9^, ^10^, ^11^

Sliding hiatal hernias (sHH; hiatal hernia type 1) are known contributors to GI CS in dogs that have been linked to GERD, EERD, and respiratory pathology, making them an example of an AeroD.^2^ Sliding hiatal hernias frequently are identified in brachycephalic dogs, with breed predispositions in French bulldogs^12^ and Chinese Shar Peis.^13^ However, sHH is not exclusive to this population. Gastroesophageal reflux disease is closely associated with sHH in people, with compromised esophageal clearance, increased transient lower esophageal sphincter relaxation, and gastroesophageal junction incompetence contributing to both typical and atypical GERD clinical signs, as well as several other aspiration‐associated respiratory syndromes (AARS).^14^, ^15^, ^16^ Brachycephalic dogs are considered to be at increased risk for GERD and AARS because of upper airway obstruction.^9^ An association between GERD and sHH has been identified in brachycephalic, but not nonbrachycephalic dogs with sHH.^17^, ^18^ The presenting CS and classification of concurrent AeroD, such as GERD, in brachycephalic compared to nonbrachycephalic dogs with sHH are unknown.

Thoracic radiographs historically have been used to detect sHH in dogs.^19^ However, conventional radiography may be poorly sensitive for the detection of sHH and accompanying episodic or dynamic AeroD, including reflux.^20^, ^21^, ^22^ Videofluoroscopic swallow studies (VFSS) represent the criterion standard for evaluation of dysphagia in veterinary medicine and may be superior for detecting and characterizing sHH and accompanying AeroD in dogs.^23^

Our primary objectives were 3‐fold. The first aim was to characterize CS of AeroD in dogs with sHH. The second aim was to compare clinical and demographic features between brachycephalic and nonbrachycephalic dogs with sHH, and the final aim was to compare thoracic radiographs and VFSS for diagnosing sHH. We hypothesized that in dogs with sHH respiratory CS would be common and associated with pathologic reflux, with CS more common and severe and pathologic reflux more frequent in brachycephalic dogs versus nonbrachycephalic dogs. We also hypothesized that VFSS would be superior to radiographs for diagnosing sHH.

## 2 MATERIALS AND METHODS

### 2.1 Case selection

Medical records of dogs presented to the veterinary teaching hospitals at Auburn University (AU‐CVM) and the University of Missouri (MU‐VHC) between 1 January 2009 and 31 December 2020 were retrospectively reviewed. Dogs were included if they were diagnosed with a sHH by radiography, VFSS, or surgery and had a complete medical record. A sHH was defined as dynamic displacement of the esophageal gastric junction past the diaphragmatic line into the thoracic cavity.^24^ In all cases, VFSS were standing and free‐feeding studies as previously described.^23^ Demographic data (age [years], breed, sex, body weight [kg], body condition score [BCS; 9‐point scale], head conformation [brachycephalic, mesocephalic, or dolichocephalic]), CS at presentation, duration of CS, and method by which sHH was diagnosed were recorded. Clinical signs, including those characteristic of brachycephalic dogs (eg, stertor) only were counted if progression prompted veterinary evaluation. Dogs were considered to be in respiratory distress if emergency intervention was required at the time of presentation. Other CS, inclusive of the degree of respiratory effort and cyanosis, were derived directly from the medical record. All radiographs and VFSS were reviewed by a board‐certified radiologist at the time of acquisition. Relevant radiographic metrics inclusive of caudal esophageal fluid (present or absent and radiographic view where observed), aerophagia (present or absent), findings consistent with aspiration pneumonia (AP; cranioventral distribution of an alveolar or interstitial pattern), and hypoplastic trachea were obtained from finalized radiographic reports where available. After enrollment, all VFSS were re‐evaluated by a single investigator with experience in interpretation of VFSS by the below‐mentioned metrics (MG).^2^, ^23^, ^25^

On VFSS, reflux margination was defined by how far cranially the contrast material traveled from the stomach. Extraesophageal reflux (EER; Video S1, Supporting Information) was defined as reflux extending beyond the esophagus to contact upper airway structures (eg, pharynx, larynx). Reflux contained within the esophagus was described as reaching the proximal third, middle third or distal third of the esophagus. Reflux extending cranially to reach the middle third of the esophagus was considered pathologic. Reflux limited to the distal third of the esophagus was considered physiologic. Aerophagia was defined as gas comprising > one‐third of the bolus volume or after filling, > one‐third of the gastric volume.^2^ Esophageal hypomotility was defined as incomplete or ineffective peristalsis.^2^ Pharyngeal weakness was defined as the presence of residual contrast material in the pharynx after an appropriately‐timed pharyngeal swallow.^26^ Elongated soft palate and nasopharyngeal collapse were diagnosed as previously described.^27^, ^28^, ^29^

### 2.2 Statistical analysis

Statistical analysis was performed by commercial statistical analysis software (SigmaPlot 12.0). Descriptive statistics were calculated where appropriate. Data for categorical variables were presented as (n) ± percentage. The normality assumption was evaluated by the Kolmogorov‐Smirnov test. All variables were non‐normally distributed. Data for quantitative variables were presented as median and interquartile range (IQR). Between group, comparisons were made using the Mann‐Whitney test and Chi‐square analysis. Correlations between demographic and clinical data and the development of respiratory CS were performed by Spearman rank correlation. To limit type 2 error, statistical comparisons were limited to CS or demographic characteristics displayed by ≥8 dogs. Clinical signs or demographic features with <8 dogs were provided descriptively. Comparisons between VFSS and radiographs for detecting sHH were performed on a subpopulation of dogs that was evaluated by both modalities. A *P* < .05 significance level was assigned in all cases.

## 3 RESULTS

### 3.1 Animals

Sixty‐seven client‐owned companion dogs met inclusion criteria: MU‐VHC (n = 52), AU‐CVM (n = 15). Twenty‐three breeds were represented (Table 1). The overall median (IQR) age at diagnosis was 2.0 years (8 months‐6.5 years; range, 1 month‐14.4 years). Thirty‐three of 67 dogs were brachycephalic and 34/67 were nonbrachycephalic. Of the nonbrachycephalic dogs, 30/34 were mesocephalic and 4/34 were dolichocephalic. Forty‐six dogs were male (23 intact; 23 castrated) and 21 were female (6 intact; 15 spayed). Median (IQR) weight was 11.6 kg (6.4‐18.8 kg; range, 2.6‐43.6 kg). Body condition scores were available for 28/67 dogs. The median (IQR) BCS was 5 (4‐5; range, 3‐8). Dogs were presented exclusively for GI CS (n = 28), a combination of respiratory and GI CS (n = 22), or exclusively for respiratory CS (n = 17). Reported respiratory and GI CS are provided in Table 2. Median (IQR) duration of CS was 3 months (1‐7.3 months; range, 1 day‐5 years).

**TABLE 1:** Number (n) of dogs of various breeds diagnosed with sliding hiatal hernia

Dogs (n)	Breed
13	French bulldogs
10	American bulldogs
9	Labrador retriever
7	Mixed breed
6	Boston terrier
3	English bulldog
2	American Staffordshire terrier, dachshund (miniature)
1	Siberian husky, Catahoula leopard dog, shih tzu, golden retriever, Yorkshire terrier, bichon frise, border collie, Australian shepherd, miniature schnauzer, German shepherd dog, dachshund (standard), Pomeranian, American cocker spaniel, Cairn terrier, Welsh corgi

**TABLE 2:** Summary of respiratory and gastrointestinal (GI) clinical signs (CS) in dogs diagnosed with sliding hiatal hernia (sHH)

Clinical signs	Brachycephalic (n)	Nonbrachycephalic (n)	Total dogs (n)
Respiratory			
Cough	7	9	16
Respiratory distress^a^*	7	1	8
Labored breathing^a^	2	5	7
Stridor	3	2	5
Collapse	2	1	3
Stertor	2	1	3
Exercise intolerance	2	1	3
Tachypnea	1	2	3
Cyanosis	0	1	1
Hiccups	0	1	1
GI			
Regurgitation	15	13	28
Vomiting	12	7	19
Anorexia	2	3	5
Abdominal pain	0	4	4
Gagging	0	2	2
Repetitive “dry” swallowing^b^	0	1	1

*Note*: Clinical signs were included in the study where there was a change in severity noted in the medical record prompting veterinary medical evaluation. Dogs may have had >1 respiratory or GI CS.

^a^ Dogs requiring emergency intervention at the time of presentation were considered to be in in respiratory distress whereas labored breathing referred to dogs that increased respiratory effort but did not require emergency stabilization.

^b^ A “dry” swallow refers to spontaneous, reflexive swallowing that occurs without eating or drinking.

*Statistically significant comparisons (*P* < .05).

### 3.2 Diagnostic imaging

Thoracic radiographs were obtained in 66/67 dogs. Videofluoroscopic swallow studies were performed in 38/67 dogs. Thirty‐seven of 67 dogs were evaluated by a combination of radiographs and VFSS, 29/67 dogs were evaluated by thoracic radiographs alone, and 1/67 by VFSS alone. Radiographic and VFSS abnormalities are presented in Table 3. Six of 12 dogs with normal thoracic radiographs presented for respiratory or mixed respiratory and GI clinical signs.

**TABLE 3:** Summary of radiographic and videofluoroscopic swallow study (VFSS) findings in dogs diagnosed with sliding hiatal hernia

Diagnostic imaging abnormalities	Brachycephalic (n)	Nonbrachycephalic (n)	Total dogs (n)
Radiographs			
sHH	16	20	36
Aspiration pneumonia^a^*	12	1	13
Unremarkable	4	8	12
Bronchial pattern	4	4	8
Esophageal fluid	6	2	8
Esophageal gas	2	3	5
Megaesophagus	0	3	3
Hypoplastic trachea	3	0	3
VFSS			
sHH	18	18	36
GER	17	15	32
Elongated soft palate	10	0	10
EER	3	1	4
Aerophagia	4	0	4
Megaesophagus	0	3	3
MSBC	0	2	2
Nasopharyngeal collapse	2	0	2
Tracheal collapse	0	2	2
Esophageal hypomotility	1	1	2
Pharyngeal weakness	1	0	1
Esophageal stricture	0	1	1

*Note*: Dogs may have had >1 finding per modality.

Abbreviations: EER, extraesophageal reflux; GER, gastroesophageal reflux; MSBC, mainstem bronchial collapse; sHH, sliding hiatal hernia.

^a^ Dogs had a radiographic diagnosis of aspiration pneumonia based on the presence of a cranioventral interstitial or alveolar pattern observed on thoracic radiographs.

*Statistically significant comparisons (*P* < .05).

A sHH was diagnosed by thoracic radiographs in 36/66 (55%) dogs and by VFSS in 36/38 (95%) dogs. Of 37 dogs that had both thoracic radiographs and VFSS, the sHH was identified by both modalities in 6. A sHH was diagnosed incidentally during an exploratory laparotomy in 1 dog presented for vomiting. This dog was evaluated by both thoracic radiographs and VFSS. For dogs having both radiographs and VFSS (n = 37), diagnosis of sHH was made significantly more often with VFSS (*P* < 0.001). A summary is provided in Table 4.

**TABLE 4:** Summary of sliding hiatal hernia (sHH) evaluation and diagnosis in 67 dogs

Modality	Dogs evaluated
Radiographs alone	29/67
VFSS alone	1/67
Radiographs and VFSS	37/67
**Modality**	**Diagnosis**
Radiographs	30/66
VFSS	30/38
Radiographs and VFSS	6/37
Other (abdominal explore)	1

*Note*: A total of 66/67 dogs had thoracic radiographs and 38/67 has videofluoroscopic swallow study (VFSS). The number of dogs evaluated alone or in combination by radiographs, VFSS, or both are provided above. The method of sHH diagnosis is also provided. Dogs listed as being diagnosed by radiographs and VFSS had a positive diagnosis on both modalities. The dog diagnosed by abdominal explore had no sHH detected on either radiographs or VFSS. A total of 36/66 dogs had a sHH diagnosed by radiographs and 36/38 by VFSS.

Reflux accompanied sHH in 32/38 dogs diagnosed by VFSS. Reflux margination was as follows: EER (n = 4), proximal esophagus (n = 14), middle esophagus (n = 9), and distal (n = 5). As such, 27 and 5 dogs were diagnosed with pathologic and physiologic reflux, respectively. Eight dogs had fluid noted in the caudal esophagus on radiographs. This fluid was presumed to be reflux based on the opinion of the radiologist and the transient nature of the finding (ie, not present on all views). In 4/8 dogs, the caudal esophageal fluid was found exclusively on the left lateral projection. The remaining 4/8 dogs had caudal esophageal fluid on the right and left lateral projections. This finding was absent on the ventrodorsal or dorsoventral projections in 8/8 dogs. Ultimately, the clinical relevance of caudal esophageal fluid on radiographs is unknown. However, 7 of 8 dogs with caudal esophageal fluid dilatation on thoracic radiographs had a VFSS and all 7 had identification of reflux on VFSS. In 6/7 of these dogs, the reflux was considered pathologic, including 3 dogs with caudal esophageal fluid identified only on the left lateral projection. Margination was as follows: proximal esophagus (n = 4) and middle esophagus (n = 2). Dogs presented exclusively with respiratory signs (*P* = .04), but not mixed CS (*P* = .09) or exclusively GI CS (*P* = .86) were more likely to have pathologic reflux identified on VFSS. Cough was not significantly more common in dogs with reflux. No correlations were identified between degree of reflux margination and presenting CS (*P* > .05).

Other diagnostic tests included functional laryngeal examinations with doxapram (n = 4), bronchoscopy with bronchoalveolar lavage (n = 3) and thoracic computed tomography (CT) scans (n = 2). Functional laryngeal examinations identified laryngeal paralysis in 3 dogs with concurrent pathologic reflux. Other findings included 22 dogs with brachycephalic obstructive airway syndrome (BOAS; Table 5), of which 16/22 had severe enough disease to warrant surgical correction at the time of surgery for the sHH. Laryngeal erythema was identified in 1 dog. Two dogs were found to have nasal tumors on head CT (n = 1) and choanal examination (n = 1), Two of 4 dogs with bronchoscopy and bronchoalvelolar lavage (BAL) were diagnosed with chronic bronchitis and 2 with septic suppurative inflammation suggestive of bacterial infection. Heavy growth of *E.coli* and *Strep. pseudintermedius* was obtained from BAL fluid from these patients. The other 2 BAL fluid cultures returned with no bacterial growth. One dog that underwent CT had evidence of bronchiectasis and peribronchovascular thickening. The second dog underwent a thoracic CT after developing a pneumothorax after surgical correction of the sHH. Atelectasis or fibrosis associated with the right middle lung lobe was identified in this dog.

**TABLE 5:** Summary of recognized aerodigestive diseases in dogs with sliding hiatal hernia

Aerodigestive disease	Total dogs (n)
Aspiration pneumonia (current or historical)	17
Pathologic reflux	27
Proximal 1/3	14
Middle 1/3	9
ERR	4
Laryngeal paralysis	3
Laryngeal collapse (nonbrachycephalic)	1 (grade 2)
BOAS	22
Elongated soft palate	17
Stenotic nares	10
Everted laryngeal saccules (grade 1 laryngeal collapse)	6
Hypoplastic trachea	3
Megaesophagus	3
Chronic bronchitis	2
Bacterial pneumonia*	2
Bronchiectasis	2
Nasopharyngeal collapse	2
Esophageal hypomotility	2
Nasal tumor*	2
Esophageal stricture	1
Pharyngeal weakness	1

*Note*: Other respiratory and digestive disorders identified during this study but not currently classified as an AeroD are denoted by *. Each dog may have had >1 problem.

Abbreviations: BOAS, brachycephalic obstructive airway syndrome; EER, extraesophageal reflux.

### 3.3 Between group comparisons: Brachycephalic versus nonbrachycephalic dogs

Brachycephalic dogs were significantly younger at diagnosis compared to nonbrachycephalic dogs (*P* < .001). Median (IQR) age was 0.9 years (0.5‐2 years) and 5.0 years (2‐9.5 years) for brachycephalic and nonbrachycephalic dogs, respectively. Brachycephalic dogs were more likely to be presented in respiratory distress compared to nonbrachycephalic dogs (Table 2; *P* = .02). Brachycephalic dogs (n = 8) also were more likely to be presented with a history of recurrent (>1 episode) AP compared to nonbrachycephalic dogs (n = 2; *P* = .04). Brachycephalic dogs were more likely to have current radiographic evidence of AP compared to nonbrachycephalic dogs (Table 3; *P* < .001). No statistically significant differences were detected between brachycephalic and nonbrachycephalic dogs for weight (*P* = .39), or BCS (*P* = .52). No significant differences were detected between brachycephalic and nonbrachycephalic dogs for type of CS at presentation (respiratory vs. GI; *P* = .14) or duration of CS (*P* = .89). Likewise, no significant differences were detected between brachycephalic and nonbrachycephalic dogs for pathologic reflux (*P* = .55), reflux margination (*P* = .80) regurgitation (*P* = .28), vomiting (*P* = .16), or cough (*P* = .62).

### 3.4 Final diagnoses

Diagnoses of concurrent aeroD and other respiratory or digestive disorders not currently classified as aeroD are provided in Table 4.

## 4 DISCUSSION

Respiratory CS, alone or in combination with GI CS, were the primary reason for presentation in 39/67 (58%) dogs with sHH. This finding emphasizes the need to consider sHH as a differential diagnosis for dogs with respiratory CS and to pursue appropriate testing. Although thoracic radiography is the most common imaging modality in general practice for evaluation of a wide variety of respiratory clinical signs, thoracic radiography was significantly less likely to diagnose sHH when compared to VFSS (55% vs. 95%, respectively; *P* < .001). Pathologic reflux of gastric contents, noted in 27/38 (71%) of dogs with sHH, was dynamically captured only by VFSS (Video S1) and might help explain the common occurrence of respiratory signs. Compared to dogs with GI CS, dogs with exclusively respiratory CS were significantly more likely to have pathologic reflux on VSS. Unexpectedly, sHH was diagnosed in similar number of brachycephalic and nonbrachycephalic dogs in our study.

Sliding hiatal hernias are clinically important AeroD that may present with a broad range of CS. Historically, the most commonly reported CS for dogs with sHH are GI CS including vomiting, regurgitation, and ptyalism.^30^ Although respiratory CS have been reported in dogs with sHH, they were in brachycephalic breeds and consistent with BOAS.^30^ The high incidence of respiratory CS in 58% of dogs in our study is consistent with a recent report finding that dogs presenting with respiratory CS frequently have occult alimentary disease.^2^ In our study, 25% of dogs were presented with exclusively respiratory CS. As such, reliance on GI CS to identify patients with sHH is likely to be poorly sensitive. Maintaining an index of suspicion of sHH as an AeroD presenting in the absence of GI CS may improve identification by prompting additional diagnostic testing.

Normal thoracic radiographs were identified in 12 dogs with sHH, including 6 that were presented with CS of respiratory disease. The lack of radiographic findings in this group may be a consequence of the relatively poor sensitivity of thoracic radiographs to detect subtle disease. In people however extrathoracic cough triggers including irritation by acid and pepsin on the larynx and pharynx, microaspiration, and cough sensitization secondary to GERD and EERD are likely contributors to respiratory CS in the absence of overt radiographic abnormalities.^31^ Cough sensitization refers to a condition in which the cough reflex is more readily induced, and has been linked to GERD and nasal disease in people.^32^ Of interest, fluid in the esophagus noted on thoracic radiography (interpreted as of unknown clinical relevance) was associated with pathologic reflux in 6/7 dogs that subsequently underwent VFSS. Follow‐up evaluation by VFSS in dogs with radiographic evidence of esophageal fluid and supportive CS thus may be indicated. After sHH, AP was the most commonly identified abnormality on thoracic radiographs. However, AP, particularly when recurrent, should be considered an indication of failed airway protection and warrants additional diagnostic testing to identify a predisposing cause.

Historically, thoracic radiography is used to diagnose sHH as well as underlying respiratory disease. However, in dynamic disease processes such as sHH, GERD, and EERD, conventional radiographs are likely to be poorly sensitive. Videofluoroscopic swallow studies represent the criterion standard for evaluation of dysphagia in dogs.^2^, ^23^ In our study, VFSS was superior to radiographs for detecting sHH. Furthermore, this modality allowed characterization of concurrent AeroD such as GERD. In our study, 84% (32/38) of dogs with sHH were found to have detectable reflux on swallow. Furthermore, VFSS allowed discrimination between physiologic and pathologic reflux based on the degree of margination. This differentiation is not possible by thoracic radiographs. However, VFSS lack the resolution to detect pulmonary parenchymal and airway‐related changes compared to thoracic radiographs. As such, a multimodal approach encompassing both VFSS and conventional respiratory imaging is warranted when evaluating dogs suspected of having sHH.^33^, ^34^

The presence of concurrent AeroD such as GERD and EERD is supported by previous studies in both dogs and people.^17^, ^35^ Although approximately 41% of heathy dogs may have GERD as detected by VFSS,^23^ in our study 27/32 (84%) were found to have pathologic reflux that traveled retrograde reaching past the distal one‐third of the esophagus. This finding is consistent with a review of studies in humans where 50%‐94% of people with sHH had evidence of reflux^35^ and in a study that identified reflux in 88% of dogs with sHH.^17^ However, the degree of margination was not evaluated. The frequency of pathologic reflux in this population may be attributed in part to loss of extrinsic pressures from the diaphragmatic crura and cranial displacement of the esophageal gastric junction.^36^ However, incomplete resolution of GERD after successful surgery for sHH in a previous study may suggest that GERD is an independent or multifactorial process occurring in combination with sHH, and treatment for reflux may be required after surgery.^17^

In our study, several concurrent conditions that increase upper airway resistance were identified. In 22/34 brachycephalic dogs, BOAS was considered severe enough to warrant surgical correction. Although less common, upper airway obstruction also was identified in nonbrachycephalic dogs, including laryngeal paralysis (n = 2), grade 2 laryngeal collapse (n = 1) tracheal collapse (n = 2), mainstem bronchial collapse (n = 2), and a nasal mass (n = 1). Unfortunately, only 4/67 dogs underwent laryngeal function examination, including the use of doxapram. As such, clear conclusions relating sHH to sources of upper airway obstruction that may be more common in nonbrachycephalic dogs (eg, laryngeal paralysis) cannot be drawn. However, previous studies have identified an association between reflux and laryngeal dysfunction in dogs.^4^, ^5^ Additional work should be done to evaluate dogs with sHH, regardless of head conformation, for evidence of airway obstruction. Likewise, the BAL fluid profiles in dogs with sHH and GERD warrant additional investigation because GERD has been implicated in the pathogenesis and progression of respiratory diseases such as asthma, bronchiolitis, interstitial pulmonary fibrosis, and chronic obstructive pulmonary disease in people.^37^, ^38^, ^39^, ^40^ Unfortunately, underutilization of BAL fluid in dogs in our study precluded evaluation of the effect of GERD on BAL fluid cytology in dogs.

Although brachycephalic dogs were not over‐represented compared to nonbrachycephalic dogs in our population, French bulldogs and American bulldogs were the most commonly identified breeds, consistent with previous studies.^11^, ^12^ Significant differences were detected between brachycephalic and nonbrachycephalic dogs for age, respiratory distress at presentation, radiographic evidence of AP, and historical recurrent AP. Interestingly, despite the severity of respiratory clinical signs in this population, brachycephalic dogs were no more likely to have pathologic reflux, increased reflux margination, regurgitation, or vomiting compared to nonbrachycephalic dogs. It is clear however that brachycephalic dogs with respiratory disease respond to treatment for reflux.^9^, ^41^ The differences in clinical severity and AP between brachycephalic and nonbrachycephalic dogs may be a result of impaired airway protective mechanisms in brachycephalic dogs. Alternatively, worsening of upper airway obstruction in brachycephalic dogs caused by pathologic reflux, regurgitation and vomiting may cause more severe respiratory CS and increased risk of AP.

Our study had some limitations. These limitations are largely related to the retrospective nature of the study. Because of small numbers of dogs presented with specific clinical signs, statistical analysis could not be performed because of increased risk of type 2 error. Not all dogs underwent a uniform or comprehensive diagnostic evaluation. For example, VFSS may not detect an elongated soft palate, which might require respiratory fluoroscopy or functional upper airway examination. A multimodal approach is needed when evaluating dogs for AeroD. Furthermore, not all dogs had both a VFSS and thoracic radiographs. As such, comparisons between modalities were made on subpopulations of dogs. Prospective studies comparing VFSS and radiographs for detecting sHH are warranted. Additionally, the influence of BOAS on the severity of respiratory clinical signs in the patient population cannot be entirely predicted. However, we attempted to mitigate this situation by not including CS for analysis unless they were listed as progressive or the reason for seeking veterinary medical attention. In dogs in which a concurrent respiratory disease was identified (eg, chronic bronchitis) the contribution of sHH, reflux, and microaspiration on airway inflammation could not be determined and thus it was unclear if these diseases represented an AeroD or were a comorbid condition.

## 5 CONCLUSION

The use of VFSS to screen patients for sHH improves detection and provides more comprehensive evaluation of concurrent AeroD compared to thoracic radiographs alone. Nonbrachycephalic dogs with sHH were commonly identified in our study. However, brachycephalic dogs presented at a younger age and with more evidence severe respiratory compromise compared to nonbrachycephalic dogs. Sliding hiatal hernias are important AeroD in dogs with the potential to cause severe CS, including those exclusive to the respiratory tract. As such, clinical awareness of the relationship between the respiratory and GI tracts is needed to maximize the identification of diseases in affected patients.

## CONFLICT OF INTEREST DECLARATION

Authors declare no conflict of interest.

## OFF‐LABEL ANTIMICROBIAL DECLARATION

Authors declare no off‐label use of antimicrobials.

## INSTITUTIONAL ANIMAL CARE AND USE COMMITTEE (IACUC) OR OTHER APPROVAL DECLARATION

Authors declare no IACUC or other approval was needed.

## HUMAN ETHICS APPROVAL DECLARATION

Authors declare human ethics approval was not needed for this study.

## Supporting information


**Video S1** An adult mesocephalic dog undergoing VFSS. Data were collected 30 fps but slowed to 5 fps for publication. The dog demonstrates extraesophageal reflux with liquid refluxate extending beyond the upper esophageal sphincter to contact structures of the upper aerodigestive tract.
